# Crafting minds and communities with Minecraft

**DOI:** 10.12688/f1000research.9625.2

**Published:** 2017-01-09

**Authors:** Benjamin C. Riordan, Damian Scarf

**Affiliations:** 1 Department of Psychology, University of Otago, Dunedin, 9054, New Zealand

**Keywords:** Minecraft, Education, Gamification, Technology, Developmental Psychology

## Abstract

Minecraft is a first-person perspective video game in which players roam freely in a large three-dimensional environment. Players mine the landscape for minerals and use these minerals to create structures ( *e.g.*, houses) and mould the landscape. But can Minecraft be used to craft communities and minds? In this opinion piece, we highlight the enormous potential of Minecraft for fostering social connectedness, collaboration, and its potential as an educational tool. We highlight the recent use of Minecraft to aid socialization in individuals with Autistic Spectrum Disorder (ASD) and promote civic engagement via the United Nations Human Settlement Program. We further provide novel links between Minecraft and recent on work on the role of social cures and community empowerment in enhancing mental health, wellbeing, and resilience.

Minecraft is a first-person perspective sandbox game – a three-dimensional, procedurally generated, Lego-like environment made up of blocks of different compounds ( Duncan, 2011; Mojang, 2016). Players mine these compounds and re-place them to create various structures or shape the landscape. Minecraft has been purchased over 100 million times and in every country in the world (incl. Antarctica; Mojang, 2016). We think Minecraft is more than a global phenomenon, and may represent a critical and historical transition; an eminently popular videogame that is highly social and collaborative ( Bainbridge, 2007; Entertainment Software Association, 2016; Granic *et al.*, 2014; Przybylski, 2014), casting doubt on the depiction of video gamers as disconnected children and adolescents ( Zimbardo & Coulombe, 2016). While early videogame research focused predominantly on the negative impacts of gaming ( Strasburger *et al.*, 2010), researchers are now starting to focus on the positives ( Lorch & Mills, 2016). We believe that Minecraft is at the forefront of this change ( Granic *et al.*, 2014; Nebel *et al.*, 2016).

Unlike other games, when played in its traditional settings, Minecraft has no aim or specific goals, which allows players the freedom to immerse themselves in their own narrative, build, create, and explore. Players can build alone, or join/create servers to play cooperatively. Given the creative nature of Minecraft and the open world environment, it is unsurprising that some have used the platform to create immersive worlds, artworks, and performances ( Bukvic *et al.*, 2014; Duncan, 2011). Importantly, Minecraft lends itself to socialization. The nature of the game has led to the formation of communities and groups that share and support creative creation. Given the move towards social and online gaming, it is unsurprising that a recent review found that videogame play is associated with social outcomes ( Greitemeyer & Mügge, 2014). But unlike other games, Minecraft may be used to actively promote socialization.

An example of this is Autcraft, a semi-private Minecraft server and online community formed around those with Autistic Spectrum Disorder (ASD; Ringland *et al.*, 2016). Those with ASD often struggle with face-to-face social interactions, but they still express a desire to connect socially. Playing Minecraft can help these players meet their social goals and gain the positive effects of socialization ( Ringland *et al.*, 2016). Although the effectiveness of Autcraft on wellbeing has not been tested, ethnographic research has suggested that the platform has successfully promoted collaboration, socialization, and community. Ensuring that individuals maintain group memberships is critical in improving health and wellbeing ( Holt-Lunstad *et al.*, 2010; Jetten *et al.*, 2012), and the Autcraft blueprint can be used to help other groups achieve this ( Osmanovic & Pecchioni, 2016). It is important to note, however, that Minecraft is likely best suited to facilitating, rather than replacing, in-person group based activities. For example, for disabled individuals who maybe largely house bound, Minecraft may provide a way to interact with fellow group members frequently; helping to keep groups together that may only meet occasionally in person. It will also be important for future studies to investigate whether Minecraft can imbue groups with the sense of belonging and acceptance that are critical to their benefits ( Scarf *et al.*, 2016a; Scarf *et al.*, 2016b).

The collaborative nature of Minecraft can also promote prosocial behavior outside the videogame context ( Gentile *et al.*, 2009; Greitemeyer & Mügge, 2014). For example, to harness the prosocial behavior Minecraft instils and to promote civic engagement, Minecraft partnered with the United Nations Human Settlement Program (UN-Habitat) to engage communities in planning urban public spaces ( Block By Block, 2016). The program provided residents with Minecraft and computer access so they could cooperatively recreate their cities and show city planners how they want their cities to look. The ubiquity of Minecraft and ease of play makes it the perfect game to promote bottom-up approaches and engage and empower communities to reimagine their city spaces ( Baba *et al.*, 2016). The program has helped communities all over the world create parks, city squares, sidewalks, seawalls, and marketplaces and offers a novel way to engage youth in shaping their environments.

Beyond social applications, Minecraft actively promotes the problem solving skills, creativity, planning, and persistence skills necessary for future success. Employers are actively recruiting gamers from online videogame leaderboards ( Carr-Chellman, 2016) and gamers’ unique skills are credited with helping scientists solve complex problems ( Cooper *et al.*, 2010). Minecraft fosters these skills as the environment requires the player to interact with unfamiliar environments, experiment, calculate, plan ahead, and develop complex mental representations to understand the world. In fact, longitudinal research suggests that videogames are related to greater problem solving skills. For example, in a high school population, strategic videogame play predicted self-reported problem solving skills, which in turn predicted better academic grades ( Adachi & Willoughby, 2013). Research has also experimentally manipulated videogame play to determine whether this correlation is causal. When undergraduates were assigned to play a strategy based game (Portal 2), relative to a group that ironically played a brain-training game (Lumosity), the strategy group displayed a significantly greater improvement in problem solving and persistence ( Shute *et al.*, 2015).

To help educators craft minds in the class room, Minecraft released an updated version, Minecraft: Education Edition in November 2016 ( Mojang, 2016). To launch off the platform laid by MinecraftEDU, the updated version includes features to facilitate classroom participation. The use of a commercial game like Minecraft in education may not only increase motivation for learning, but allow students to take a more active role in their education. Already, a number of educators have taken advantage of the collaborative nature of Minecraft to plan immersive lessons, and homework in subjects such as math, earth and ocean science, chemistry, molecular biology, and history (for a review see Nebel *et al.*, 2016). Outside the classroom, platforms already exist to help students explore geology or the stories behind famous art ( British Geological Society, 2017; Tate Modern, 2017). Although there are few studies on using Minecraft in the classroom, educators have consistently reported that Minecraft has improved interest and motivation for learning ( Nebel *et al.*, 2016). For example, to measure the effectiveness of Minecraft as a teaching tool, one 7 ^th^ grade class was taught with Minecraft and another with traditional lecture based learning. Both groups showed improvement, however, post-tests indicated those who had been taught with Minecraft performed significantly better ( Wang & Towey, 2013). Unfortunately, this is one of the few studies to use an adequate control group to test the effectiveness of Minecraft in the classroom. Despite this, we believe that the functions added to Minecraft Education Edition will allow Minecraft to become a mainstay in education and help craft minds.

While more empirical data is needed, the use of Minecraft to foster socialization, engage and empower communities, and enhance students’ interest in education and creation suggests that Minecraft is crafting minds and opening a new chapter in video game research.
