# Supplementary material for: Crafting minds and communities with Minecraft
Source: F1000Res. 2017 Jan 9;5:2339. Originally published 2016 Sep 19. [Version 2] doi: 10.12688/f1000research.9625.2 (PMC5381616; doi:10.12688/f1000research.9625.2)
Supplement: Peer review history

# Peer review: Referee response for version 2

**DOI:** 10.5256/f1000research.11434.r21361

**Published:** 2017-03-30

**Authors:** Ascensión Hernández-Encinas

**Affiliations:** 1 Department of Applied Mathematics, University of Salamanca, Salamanca, Spain

This paper presents the use of Minecraft by individuals with ASD, with others. From our point of view there are some missing aspects that could improve the paper. This is only an introduction about the use of the program in the classroom. This kind of paper should include more specific aspects of the use of that tool in this adequate environment. Some specific results would be appreciated, to test the suitability of Minecraft.

I have read this submission. I believe that I have an appropriate level of expertise to confirm that it is of an acceptable scientific standard.

# Peer review: Referee response for version 2

**DOI:** 10.5256/f1000research.11434.r19056

**Published:** 2017-02-01

**Authors:** Mark Lorch

**Affiliations:** 1 Department of Chemistry, University of Hull, Hull, UK

I would like to thank the authors for addressing my earlier comments and adjusting their manuscript accordingly. The latest version presents a clear overview of some of the ways that Minecraft has been used in educational and provides context though reference to existing video game research. 

 The additional insight into the authors' opinions are welcomed. 

 I am happy to now approve the paper.

I have read this submission. I believe that I have an appropriate level of expertise to confirm that it is of an acceptable scientific standard.

# Peer review: Referee response for version 1

**DOI:** 10.5256/f1000research.10369.r17059

**Published:** 2016-10-24

**Authors:** Mark Lorch

**Affiliations:** 1 Department of Chemistry, University of Hull, Hull, UK

The article is a very brief introduction to some uses of Minecraft outside of its core gamer  base. The authors highlight some interesting projects, but do not dicuss them at any length. 

 Beyond providing a handful of examples no real attempt has been made to discuss how Minecraft can or might be used to craft minds and communities. Nor do the authors express much of an opinion on the topic.

 Furthermore some fundamentals and high profile attempts to use Minecraft as an educational tool projects have been overlooked. For example the authors imply that the first versions of Minecraft designed for educational uses was launched in September 2016. However MinecraftEDU has been available since 2011. Other educational projects such work by the Royal Geological Society, the Tate Modern are not mentioned.

 The article does cover a very interesting and rich area for which I would like to see a comprehensive review or opinion piece. This article is a good start however much more depth, both in terms of examples, discuss and opinion is needed.

I have read this submission. I believe that I have an appropriate level of expertise to state that I do not consider it to be of an acceptable scientific standard, for reasons outlined above.

# Response

**Published:** 2017-01-03

**Authors:** Damian Scarf

We thank the reviewer for their thoughts and suggestions and address some of their comments below. We hope that our amendments to the manuscript meet their concerns.

1. *The article is a very brief introduction to some uses of Minecraft outside of its core gamer base. The authors highlight some interesting projects, but do not discuss them at any length.* *Beyond providing a handful of examples no real attempt has been made to discuss how Minecraft can or might be used to craft minds and communities. Nor do the authors express much of an opinion on the topic.*

Unfortunately, we are limited by the amount we can say due to a tight word limit. The aim was not to discuss these points at great length but to provide a succinct and accessible overview of the promise of Minecraft in a number of areas. Very few studies using Minecraft have used adequate control groups, so where possible we have drawn on other videogame literature that has used more robust methodologies. At this stage we believe the research is not fleshed out enough for a large scale review or meta-analysis.

 However, we agree that we may be light on an opinion and make our thoughts more explicit. We now draw on social psychology literature to make more of an argument for the use of Minecraft to help craft communities and some potential limitations.

  

1. *Furthermore some fundamentals and high profile attempts to use Minecraft as an educational tool projects have been overlooked. For example the authors imply that the first versions of Minecraft designed for educational uses was launched in September 2016. However* *MinecraftEDU* *has been available since 2011. Other educational projects such work by the* *Royal Geological Society* *, the* *Tate Modern* *are not mentioned.*

We thank the reviewer for these suggestions and have added in more high profile uses of Minecraft.

 We agree we have made an error in implying that Minecraft Education Edition is the first being used for education. This was not our intention. We make this point more explicit.

  

1. *The article does cover a very interesting and rich area for which I would like to see a comprehensive review or opinion piece. This article is a good start however much more depth, both in terms of examples, discuss and opinion is needed.*

 

 We agree that this is an important area and are excited to see some more results. We hope more pieces like these will compel other researchers to run randomised controlled trials to address some of the gaps in the literature. We believe Minecraft can and should be more than just a ‘cool’ way to present education.
